# Biogeographical Patterns of Earwigs in Italy

**DOI:** 10.3390/insects14030235

**Published:** 2023-02-26

**Authors:** Simone Fattorini

**Affiliations:** Department of Life, Health and Environmental Sciences, University of L’Aquila, Via Vetoio, 67100 L’Aquila, Italy; simone.fattorini@univaq.it

**Keywords:** beta diversity, biogeography, glacial refugia, macroecology, Mediterranean, Dermaptera, peninsula effect, Pleistocene

## Abstract

**Simple Summary:**

Italy plays a central role in the research on Europe’s biogeography because of its position in the middle of the Mediterranean, which is a global hotspot of diversity. This study investigated how present patterns of earwig species richness and composition in Italy are affected by climatic, geographical, and historical factors. Earwig richness does not decrease from the base to the tip of the Italian peninsula, which contrasts with the so-called ‘peninsula effect’. However, richness does not increase southward either, suggesting that southern regions did not play a crucial role as a refuge during Pleistocene glaciations. Inter-regional similarities in species composition between regions is more influenced by their geographical proximity than climatic similarity, although richness is positively correlated with precipitation, in accordance with earwig preferences for humid conditions. The similarity in species composition with central European fauna decreases southward, indicating possible exchanges between central Europe and Italy. The majority of the earwigs of the Italian fauna are either widespread across Europe and the Palearctic, or confined to the main Italian mountain ranges, that is, the Alps and the Apennines. The isolation of ancient earwig populations on mountains resulted in the development of a high proportion of endemics, making the Italian earwig fauna one of the richest in Europe.

**Abstract:**

Placed in the center of the Mediterranean biodiversity hotspot, Italy plays a central role for the study of Europe’s biogeography. In this paper, the influence of climatic, spatial, and historical factors on current patterns of variation in earwig species richness and composition is investigated. The Italian earwig fauna is mainly composed of species which are either widely distributed in Europe and the Palearctic region or that are endemic to the Alps and the Apennines. Variation in species richness does not follow any obvious geographical patterns, but a positive influence of precipitation on richness is consistent with earwig preferences for humid climates. European mainland territories did not contribute substantially to the current biodiversity of Italian earwigs, which explains the lack of a distinct peninsula effect, although a southward decrease in similarity with the central European fauna was observed. However, southern areas did not exert a pivotal role during Pleistocene glaciations in determining current patterns of species richness. Variation in species composition among Italian regions can be mostly explained by geographical proximity, while climatic differences and historical (paleogeographical and paleoecological) events seem to have played a minor role. However, the isolation of ancient earwig stocks on Italian mountains led to the origin of a relatively large number of endemics, which makes the Italian earwig fauna one of the richest in Europe.

## 1. Introduction

Earwigs (Dermaptera) are a small order of hemimetabolous insects comprising about 2000 known species worldwide [1]. Most earwig diversity is concentrated in the tropical regions, whereas relatively few species inhabit the temperate zone of the Northern Hemisphere [2]. This has been interpreted as a result of the Gondwanan origin of earwigs, whose colonization of the Northern Hemisphere was largely hampered by unfavorable climate conditions and geological processes, such as the rising of the Himalayan range [3].

About 90 species of earwigs are known from Europe, 27 of which occur in Italy, which ranks second for the number of species among the European territories (the richest territory is Spain, which hosts some 30 species) [4,5]. Information on the taxonomy and distribution of Italian earwigs is very accurate, which makes the Italian fauna one of the best known among the European countries [4,6,7,8,9,10,11,12,13,14,15,16,17]. This fortunate circumstance makes it possible to use earwigs as a target group to investigate biogeographical patterns in Italy. Additionally, due to its geographical location in the middle of the Mediterranean Basin, one of the global biodiversity hotspots [18,19], and its extraordinarily complex geological history, Italy assumes a prominent position in the study of the European biogeography [20,21,22,23].

Following previous research on the Italian biogeography [22,24], in this study the role of historical and environmental factors in shaping earwig biogeographical patterns on the Italian peninsula and adjacent large islands (Sicily, Corsica, and Sardinia) is investigated. To this end, the potential role of climatic factors and the geographical position as drivers of patterns of species richness and spatial turnover (species replacement between regions) is examined, making use of the increased power offered by concurrently testing numerous hypotheses [25]. Specifically, the following predictions are tested:

Prediction 1a. As a result of the role of the southern regions as refugial areas during the Pleistocene glacials, earwig species richness is expected to increase southward, thus following the ‘latitudinal gradient’, one of the most prominent patterns of biodiversity [2,21,26,27,28,29,30,31,32,33,34,35,36,37,38,39,40,41]. This is because during the Pleistocene glacials, most of central and northern Europe was covered with ice, and their current faunas are a result of post-Pleistocene range expansions from southern refugia. Within the Italian peninsula, this should translate into a negative relationship between latitude and richness, as documented, for example, in tenebrionid beetles [22].

Prediction 1b. According to the ‘peninsula effect’ [42,43], species richness is expected to decrease southward if the Italian earwig fauna mainly originated from the colonization from European mainland, a pattern opposite to that of Prediction 1a. This pattern was observed for the odonates [24], which, after deglaciation, moved northward, concentrated in central European areas, and then moved southward again [24].

Prediction 2. Rainfall is expected to be a prominent climatic correlate of earwig diversity because of the general preferences of these insects for wet places [1], as already observed for odonates, which depend on freshwater for completing their life cycle [24]. By contrast, a negative correlation was found for the Italian tenebrionids, which are mainly thermo-xerophilous insects [22].

Prediction 3. Species composition differs among regions because of the action of climatic filtering on common species pools [44]. Under this hypothesis, inter-regional dissimilarities in species composition (β-diversity) should correlate with climate [41], as already observed for tenebrionids [22] and odonates [24].

Prediction 4a. Spatial patterns of species turnover are entirely due to random processes according to the geographical position of the regions. According to neutral models, species distributions result from stochastic population dynamics and spatially-constrained dispersal [45]. These stochastic processes would generate a distance decay of similarity independently from ecological differences among areas [41].

Prediction 4b. Variation in species composition between regions reflects historical (paleogeographical and paleoecological) factors beyond their geographical proximity (as postulated by Prediction 4a) and ecological similarities (such as similarity in climatic conditions, as postulated by Prediction 3). In this case, compositional similarities among regions should reflect their history (such as the effects of glacials), as already observed for tenebrionids [22] and odonates [24]. This would produce similarities among regions that are not explainable by position and climate. Additionally, if the Italian fauna has been largely influenced by historical events, a high incidence of endemics is expected as a result of ancient processes of allopatric speciation due to the effects of paleogeographical and paleoecological changes on the distribution of ancient populations.

Prediction 5. Biogeographical similarities between Italian regions and adjacent countries reflect post-glacial colonization trajectories. Previous research revealed multiple colonization trajectories, both in tenebrionids [22] and odonates [24]. For tenebrionids, during the Pleistocene glacials, southern Italian regions (which have high levels of endemicity) acted as an important refugial center for thermophilous species of North African or east Mediterranean origin, while cold-adapted species coming from northern areas (Central Europe) dispersed into central and southern Italy through the main mountain ranges [22]. For the odonates, the Italian area may have played an important role as a refugial center, but with deglaciation, these insects moved towards central European areas, from which they re-colonized the Italian peninsula, leading to a pattern of southward decreasing biogeographical similarities with European faunas [24].

Thus, we can expect: (1) high similarities of Northwestern Italian regions with the French fauna through the Provencal area; (2) southward decreasing similarity with the Central European fauna; (3) high similarities of Northeastern Italian regions with Balkan territories through the Karst Plateau; and, finally, (4) high similarities of Southern regions with the Northern African fauna because of the possible persistence of a pre-Pleistocene (Tertiary) fauna and post-Pleistocene immigration of thermophilous species.

## 2. Materials and Methods

### 2.1. Data Collection

**Figure 1:**
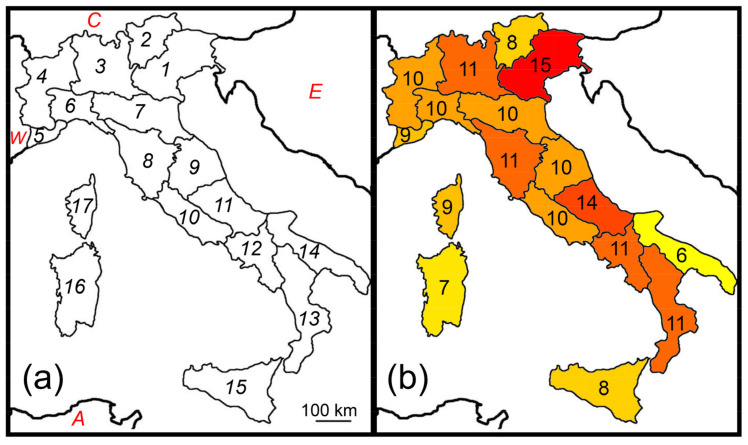
Species richness patterns of Italian earwigs. (**a**) Italian natural regions (numbered from 1 to 17) and major adjacent areas (W: Western Europe; C: Central Europe; E: Eastern Europe; A: Northern Africa); (**b**) Number of total earwig species in each region.

As in previous papers [22,24], both the Italian peninsula and the three major adjacent islands (Sicily, Sardinia, and Corsica) were considered in this study. Mainland Italy was subdivided into 17 geographical regions (Figure 1a) as proposed by Baroni Urbani et al. [46] and later used by Fattorini [22,24]. The use of regions instead of grids may be preferable to avoid problems of irregular sampling. Moreover, regional data are more accurate and comprehensive than point records [47,48,49], and are proved robust to the violation of constant grain size [50]. In general, this type of data is particularly appropriate to disclose biogeographical patterns and the underlying mechanisms [37,39,40,41,51,52,53].

The taxonomy was according to Fontana et al. [4]. A matrix of species presence/absence in each geographical region and adjacent areas (Table S1) was compiled by assembling data from literature sources [4,6,8,9,10,12,13,14,15,16,17,54,55,56,57,58]. Introduced species were excluded.

### 2.2. Statistical Analyses

Before analysis, spatial autocorrelation between richness values and region centroids was tested by using the Moran *I* index, which showed no significant spatial autocorrelation (*I* = −0.077, *p* = 0.839).

Predictions 1a and 1b were tested by correlating (Pearson product-moment correlation coefficient) species richness with the centroid latitude of mainland regions.

Prediction 2 was tested by evaluating the importance of the following climatic variables in determining species richness: average total annual precipitation (Pmean), average annual temperature (Tmean), mean minimum temperature (Tmin), mean maximum temperature (Tmax), and yearly temperature difference (ΔT = Tmax − Tmin) [22,24,39,40]. Geographical parameters (i.e., area, latitude, and longitude of regions) were also considered. Geographical and climatic data were taken from Fattorini [22]. The importance of these variables in explaining species richness was tested using a multimodel inference approach by running models representing every possible combination of explanatory variables and then ranking them according to the values of the corrected Akaike Information Criterion (AICc). Models with a difference in the AICc values lower than two were averaged using both full and conditional averages. In the full average, regression coefficients for variables that are not included in a given model are set to zero, whereas conditional average only averages over the models where the parameter appears.

Predictions 3, 4a, and 4b were tested by correlating inter-regional biogeographical distances with geographical and climatic distances. For this purpose, biogeographical distances between regions were expressed using the overall ß-diversity (ßsor; that is 1-Sørensen index of similarity), the pure turnover component (ßsim; that is 1-Simpson index of similarity, which expresses compositional differences independently from the influence of nestedness), and the nestedness component (ßnest; that is ßsor- ßsim, which quantifies the part of the compositional change caused by ordered species loss) [59,60]. Geographical distances were calculated as distances between centroids. Climatic distances were calculated as Euclidean distances for the climatic variables reported above after standardization. Matrices were correlated using Mantel tests (to evaluate the effect of either geographical position or climatic conditions on biogeographical distances) and partial Mantel tests (to evaluate the effect of geography controlling for the effect of climate, and to evaluate the effect of climate controlling for the effect of geography) [22]. Additionally, biogeographical relationships between regions expressed by Sørensen and Simpson indexes were represented by using Non-Metric Multidimensional Scaling (NMDS), an ordination technique that allows the representation of dissimilarities among areas in a two-dimensional space, and which is therefore particularly effective at disclosing multiple relationships in biogeographical analyses [24,61,62]. The aim of NMDS is to find a spatial configuration of points in which the distances between pairs of points in the configuration matches as well as possible the original dissimilarities between the pairs. To this end, NMDS starts with random initial conditions and iteratively seeks new solutions which are evaluated using a measure of goodness of fit called stress (lower values indicating better fit). Procrustes distances were used to compare solutions until a minimum stress value was obtained. In the two-dimensional representation, the axis with the highest variance was standardized between 0 and 1, and the other axis was rescaled according to the first one. Next, the colors blue, green, yellow, and red were assigned to the four corners, and each area received an RGB (red, green, blue) color according to its position in the two-dimensional space.

Prediction 5 was tested by considering the distribution of each species of the Italian fauna in the following adjacent areas: Western Europe (fauna of France), Central Europe (faunas of Austria, Switzerland, and Germany), Eastern Europe (faunas of Slovenia, Albania, Bosnia, Croatia, Herzegovina, Montenegro, and mainland Greece), and Northern Africa (Tunisia and close areas). Next, the similarity (Sørensen and Simpson indexes) between these areas and the Italian regions was calculated and mapped. The Pearson correlation coefficient was used to test for correlation between these biogeographical similarities and latitude (use of Moran *I* tests showed non-significant spatial autocorrelations).

Finally, to describe the biogeographical composition of the Italian earwig fauna, species were assigned to chorotypes (i.e., groups of species with similar distributions [63,64]) following the nomenclature proposed by Vigna Taglianti et al. [65].

All analyses were performed in R 4.1.3 software [66] using the following packages: ape 5.6–2 [67,68] and geosphere 1.5–14 [69] (for Moran *I* tests), MuMIn 1.46.0 [70] (for multimodel inference analyses), vegan 2.6–2 [71] (for Mantel tests and NMDS) and recluster 2.9 [72,73] (for NMDS).

## 3. Results

Earwig species richness did not show a latitudinal pattern (correlation between number of species and latitude: *r* = 0.026, *p* = 0.929, Figure 1b). However, very low values were observed in Apulia (region 14), Sicily (region 15), and Sardinia (region 16), which are particularly arid regions, and average total annual precipitation (Pmean) was the most important factor influencing earwig species richness in Italy (Table 1).

Thus, in contrast with Prediction 1b, species richness did not decrease southward in response to decreasing rainfall, although precipitation was an important predictor of richness, as expected according to Prediction 2. A possible positive influence of temperature appeared when the conditional average was used.

**Figure 2:**
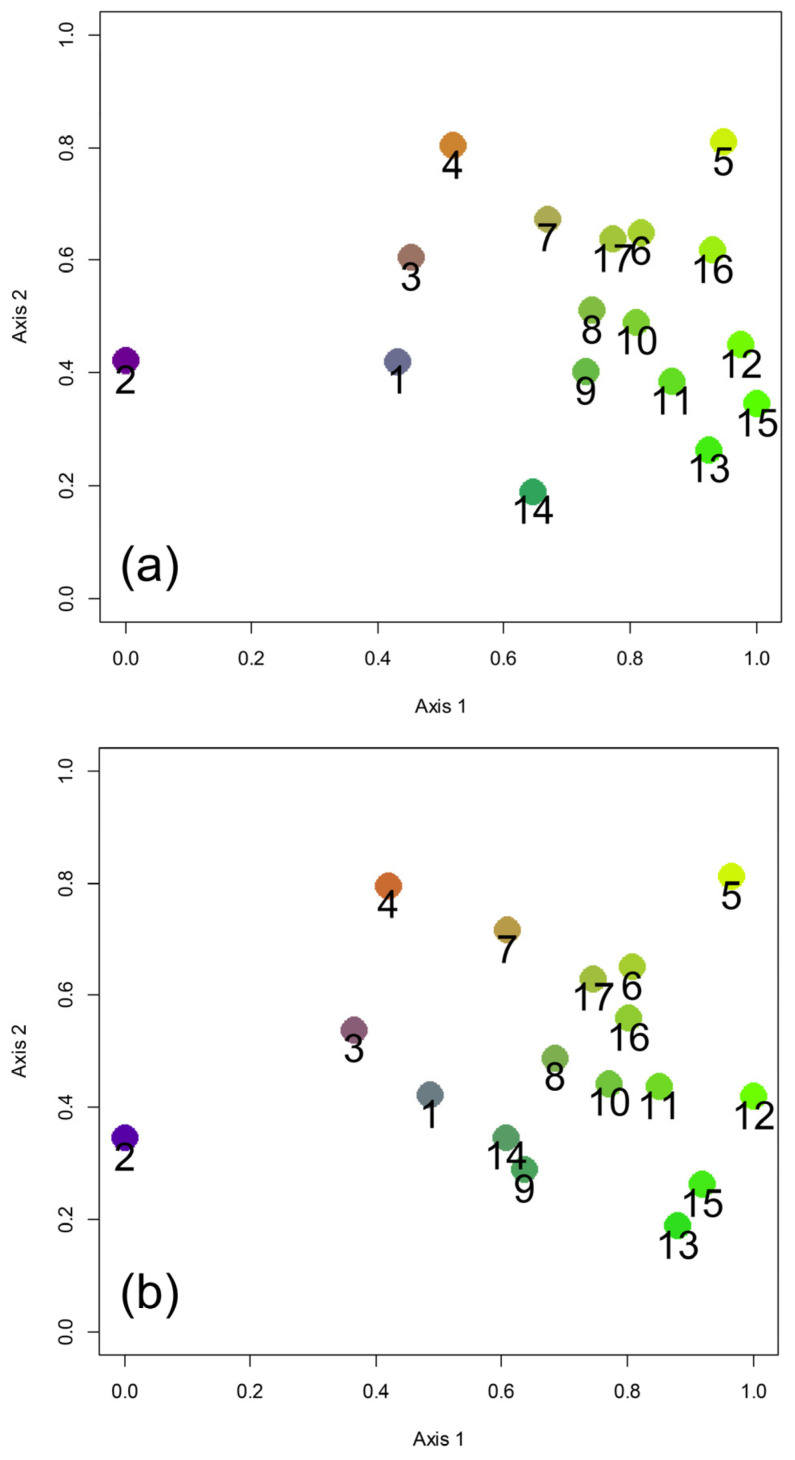
Biogeographical relationships among Italian regions expressed using Non-Metric Multidimensional Scaling (NMDS) with the Sørensen index of similarity (**a**, stress: 0.107) and Simpson index of similarity (**b**, stress: 0.128) based on earwig species composition. Regions are numbered as in Figure 1a.

The NMDS with Sørensen index (Figure 2a) showed a strong separation of region 2 (Trentino—Alto Adige) from all other regions. Alpine regions 1, 3, and 4 formed a distinctly recognizable group. The regions south of the Po River formed a complex group in which the Tyrrhenian regions (regions 8, 9, 10, 11, and 12) appeared to be closely associated. Apulia (region 14) occupied a very distinct position, while the three islands appeared close to the adjacent Italian mainland areas: Sicily (region 15) was close to Calabria (region 13) and Campania (region 12); Sardinia (region 16) was close to regions facing the north-western sector of the Tyrrhenian Sea (regions 5 and 6); and Corsica (region 17) was close to Liguria (region 6).

The results of NMDS with Simpson index (Figure 2b) were very similar to those obtained with the Sørensen index in showing: (1) a strong separation of region 2 (Trentino—Alto Adige) from all other regions, (2) the presence of a group of Alpine regions (regions 1, 3, and 4), and (3) complex relationships between the regions south of the Po river and the three islands. Sicily (region 15) resulted to be very close to Calabria (region 13), whereas Sardinia (region 16) was very close to Corsica (region 17) and Liguria (region 6). These results indicate a major role exerted by current geographical settings (the position of the main mountain ranges, i.e., the Alps at north and the Apennines at south, and geographical proximity) more than by paleogeographical and paleoecological history, therefore being contrary to Prediction 4b.

Sørensen distances correlated (Mantel tests) with both geographical (*r* = 0.479, *p* < 0.001) and climatic (*r* = 0.482, *p* = 0.005) distances. Similarly, Simpson distances were correlated (Mantel tests) with both geographical (*r* = 0.383, *p* = 0.003) and climatic (*r* = 0.355, *p* = 0.028) distances. No correlation (Mantel tests) was found between the nestedness component and geographical distances (*r* = 0.046, *p* = 0.310), and between the nestedness component and climatic distances (*r* = 0.106, *p* = 0.264).

Partial Mantel tests (Table 2) showed that correlations between biogeographical dissimilarities (calculated as either Sørensen or Simpson index) and geographical position were significant even after controlling for climate, whereas correlations between biogeographical dissimilarities and climate were not significant after controlling for geographical position. This indicates an importance of the geographical position independently from the effect of climate (thus supporting Prediction 4a), whereas (in contrast with Prediction 3) there was no relevant influence of climate on biogeographical dissimilarities independently from the geographical position.

**Figure 3:**
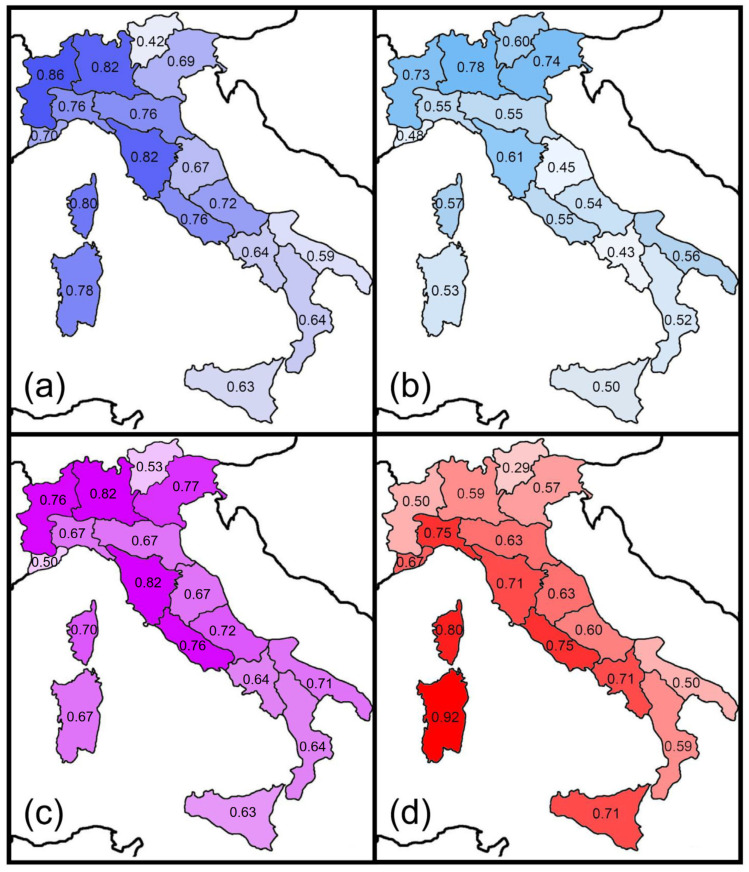
Spatial variations in the Sørensen index of similarity between Italian regions and adjacent major areas for earwigs: (**a**) Western Europe; (**b**) Central Europe; (**c**) Eastern Europe; (**d**) Northern Africa.

**Figure 4:**
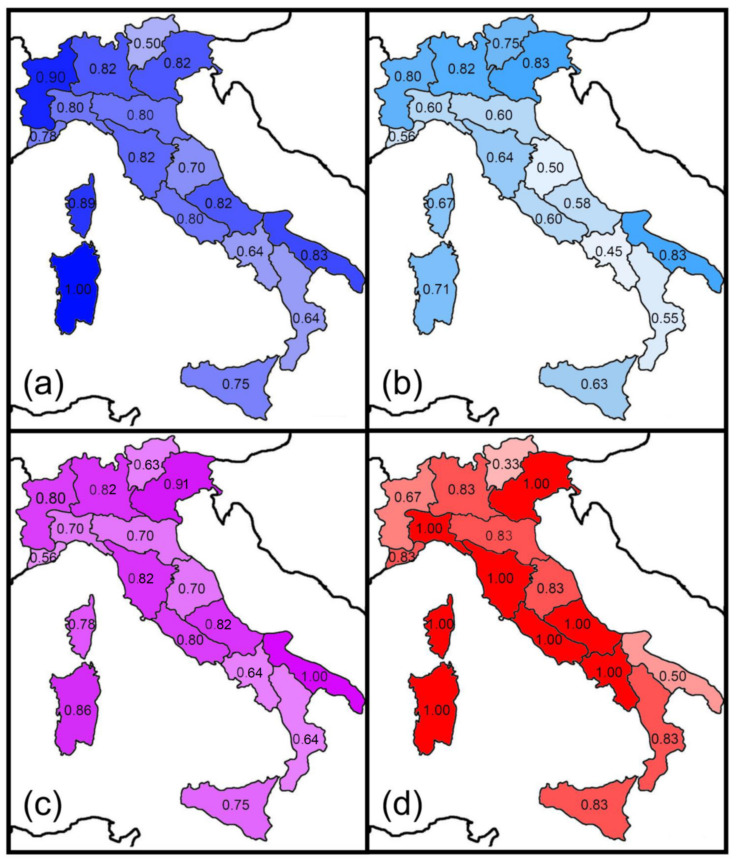
Spatial variations in the Simpson index of similarity between Italian regions and adjacent major areas for earwigs: (**a**) Western Europe; (**b**) Central Europe; (**c**) Eastern Europe; (**d**) Northern Africa.

The similarity between Italian regions and adjacent areas expressed by the Sørensen and Simpson indices pointed to some recognizable geographical patterns (Figure 3 and Figure 4).

The highest values of the Sørensen index for the Western European fauna are centered in a western sector, and were recorded in the peninsular regions facing the northern and central part of the Tyrrhenian Sea (regions 3, 4, 5, 6, 8, and 10) as well as in Sardinia (region 16) and Corsica (region 17) (Figure 3a). High values of the Sørensen index for the Central European fauna were recorded in the regions of the Alpine arch and in Corsica (Figure 3b). Similarities with the Eastern European faunas did not show any obvious pattern (Figure 3c). Finally, Sardinia (region 16) had the highest similarity value with the North African fauna (Figure 3d). Using the Simpson index, the highest values for the Western European fauna were recorded in Sardinia (region 16), Corsica (region 17) and region 4 (which includes the Western Alps) (Figure 4a). The highest values for the Central European faunas were recorded in the Alpine regions (Figure 4b), whereas those for the Eastern fauna were observed in region 1 (which includes the Eastern Alps) and region 14 (Apulia, the easternmost Italian region) (Figure 4c). Values for the North African fauna were rather uniformly high because most of the few species included in this category are very widespread (Figure 4d). For the Central European fauna, Sørensen similarities decreased southward, whereas no significant correlation was found between latitude and similarity for the Western European and the Eastern European fauna (Table 3). A slightly negative correlation was found between the latitude and Sørensen similarities for the African fauna (Table 3). No significant correlation was found for the Simpson index. These results are in partial agreement with Prediction 5.

**Figure 5:**
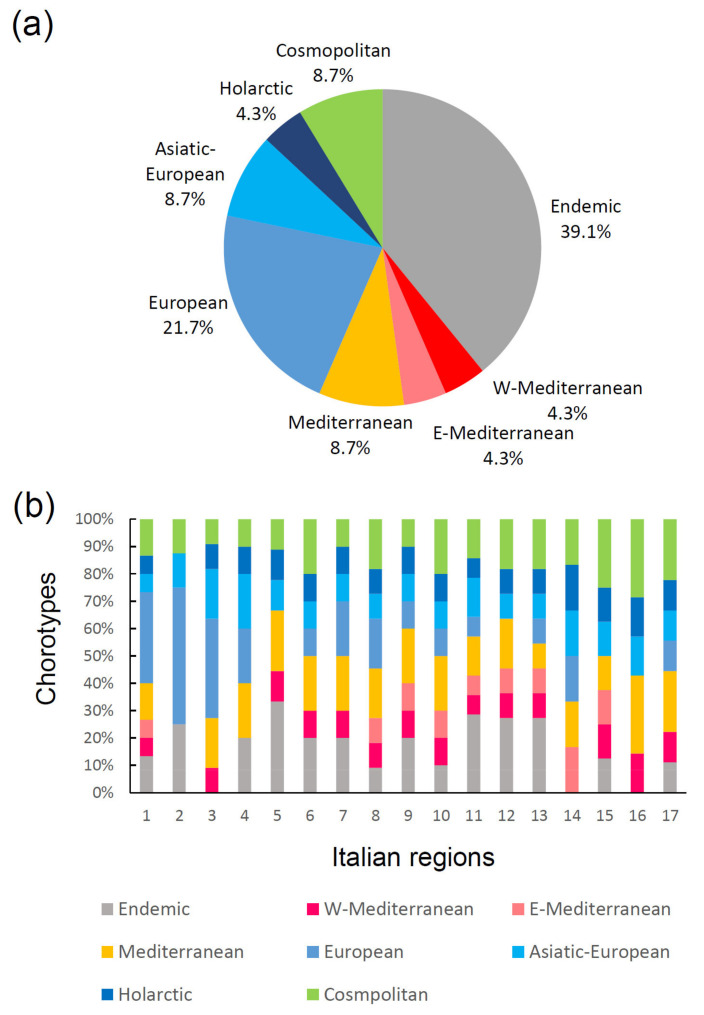
Chorological composition of the Italian earwig fauna: (**a**) proportion of earwig chorotypes for the whole fauna; (**b**) proportions of chorotypes in each Italian region. Regions are numbered as in Figure 1a.

In general, the Italian earwig fauna shows three main biogeographical groups of species (Figure 5): (1) species with broad distributions in Europe and the Palearctic region (Cosmopolitan, Holarctic, Asiatic-European, and European chorotypes): (2) species distributed in the Mediterranean area (W-Mediterranean, E-Mediterranean, and Mediterranean chorotypes), and (3) endemic species (i.e., species occurring only in Italy, including the islands). Endemics represent a conspicuous component of the total fauna (about 40%), and are present in all regions except Lombardy (region 3), Apulia (region 14) and Sardinia (region 16). The high incidence of endemics suggests a certain role of historical factors as drivers of cladogenetic processes, in accordance with Prediction 4b.

## 4. Discussion

Italian earwigs do not show any distinct latitudinal gradient in species richness, which contrasts with previous findings showing either an increasing diversity southward (as observed in dung beetles and tenebrionids [22,74]), nor an increasing diversity northward (as found in birds, small mammals, ondonates, carabid beetles, hydradephagan beetles, and ants [24,42,75]). Thus, earwigs follow neither Prediction 1a (‘latitudinal gradient’) nor Prediction 1b (‘peninsular effect’). This unexpected result may be explained by the possible contrasting effects of the spatial distribution of temperatures (which increase southward) and humidity (which increases northward) [76]. In general, earwigs tend to prefer warm and humid climates. In Italy, however, the warmest areas (located in the southern parts of the country) are also the driest, whereas the most humid regions (located in the north) are also the coldest, as shown by the distribution of Köppen-Geiger climate types [76]. Although inter-regional biogeographical relationships were correlated with climatic dissimilarities, this correlation disappeared when controlling for geographical position, which contrasts with the hypothesis of a primary role exerted by climatic conditions in shaping variations in species composition, as postulated by Prediction 3, but supports a strong influence of geographical position independently from climate, as postulated by Prediction 4a. This indicates that earwig species distributions in Italy were more profoundly shaped by geographical barriers/connections than by climate. The results of the ordination analysis (NMDS) indicate: (1) a major division between the Alpine and the Apennine faunas; (2) a distinct separation of region 2 (Trentino—Alto Adige, which is the coldest region) from all other regions; (3) a high similarity between Sicily (region 15) and the adjacent mainland (Calabria, region 13); (4) a high similarity between Corsica (region 17) and the adjacent mainland (Liguria, region 6); (5) a strong similarity between Sardinia (region 16) and both Corsica and Liguria. These patterns point to the role of the main geographical characteristics of the study area in shaping biogeographical similarities, whereas the possible importance of historical factors (Prediction 4b) seems to be secondary. Even the similarity between Corsica and Sardinia (already observed in other insects, such as butterflies [48], tenebrionids [22], carabids [46], and chrysomelids [46], but not in odonates [24]), and their similarity with the adjacent mainland, seem to be more easily interpretable for earwigs as a result of geographical proximity than as evidence of their shared paleogeographical history as a microplate (Corsardinia: Corsica + Sardinia) detached from the Provencal area [22]. However, it is important to note that about 40% of the native earwig fauna of Italy (including Sicily, Corsica, and Sardinia) is endemic to this area (and this proportion would be even higher considering that the distribution of *Chelidurella pseudovignai* Kočárek & Kirstová, 2020 and *C. mutica* (Krauss, 1886) out of Italy is restricted to areas close to the borders of the study area), and relatively high proportions of the endemic species are found in most of the regions. The earwigs endemic to Italy can be classified into two main groups whose distributions correspond to the two main mountain ranges: the Alpine endemics (*Chelidura aptera* (Megerle in Charpentier, 1825), *Chelidurella poggii* Capra, 1982, and *C. vignai* Galvagni, 1995) and the Apennine endemics (*Chelidurella caprai* Vigna Taglianti, 1993, *Pseudochelidura galvagnii* Vigna Taglianti, 1999, *P. orsinii* (Gené, 1833), *Forficula apennina* Costa, 1881, and *Forficula silana* Costa, 1881) [4,8], which is consistent with the recognized importance of mountain ranges for cladogenetic processes in these animals in Europe [77].

In agreement with Prediction 5 and findings for other groups [22,24], Italian earwigs showed a southward decline in their similarity with the Central European fauna and an opposite pattern for their similarity with the North African fauna. However, in contrast with Prediction 5, there were no clear relationships with the Western and Eastern European faunas. Additionally, no correlation was found when the pure turnover component (Simpson index) was used. Looking at the distribution of the species in the Italian territory, it appears that the Italian fauna is composed of a mixture of species with very restricted distributions on one hand (with many endemics), and widely distributed species on the other hand (with species widely distributed in Europe, the Palearctic region, or even broadly). This suggests that the Italian territory was invaded by stocks of species with high dispersal capabilities without distinct colonization routes. However, some ancient events of colonization resulted in the isolation on mountain areas of the ancestors that evolved into the current endemic species.

## 5. Conclusions

The Italian earwig fauna is mainly composed of either species which are widely distributed in Europe and the Palearctic region, or species endemic to the two main mountain ranges, that is, the Alps and the Apennines. Variation in species richness does not follow any obvious geographical pattern, but a positive influence of precipitation on richness is consistent with the ecological preferences of these insects for humid climates. Contrary to what could be expected on the basis of the ‘peninsula effect’, earwig richness did not decrease southward along the Italian peninsula, thus indicating that European mainland territories did not contribute much to the current biodiversity of Italian earwigs, at least not through distinctly recognizable pathways (with exception of a certain southward decrease of similarity in species composition with the central European fauna). However, an opposite pattern of increasing diversity southward was not observed either, which indicates that the possible refugial role of southern areas during Pleistocene glaciations did not exert a pivotal role in determining current patterns of species richness in Italy. The variation in species composition among Italian regions can mostly be explained by geographical proximity, while climatic differences and historical (paleogeographical and paleoecological) events seem to be less important. However, the isolation of ancient earwig stocks on Italian mountain regions led to the origin of a relatively large number of endemics, which makes the Italian earwig faunas one of the richest in Europe.

## Figures and Tables

**Table 1:** Results of multimodel selection for the influence of geographical and climatic variables on earwig species richness in Italy. SE: standard error, Area: surface of region (km^2^), Pmean: average total annual precipitation (mm), Tmean: average annual temperature (°C), Tmax: mean maximum temperature (°C).

Parameter	Estimate	SE	*p*-Value
Intercept	−6.101	9.184	0.519
Full average			
Area	3.619 × 10^−5^	5.741 × 10^−5^	0.541
Pmean	0.019	0.005	<0.001
Tmax	0.143	0.181	0.441
Tmean	0.048	0.129	0.715
Latitude	−0.047	0.479	0.755
Conditional average			
Area	9.896 × 10^−5^	5.291 × 10^−5^	0.089
Pmean	0.019	0.005	<0.001
Tmax	0.306	0.141	0.048
Tmean	0.305	0.165	0.092
Latitude	−0.379	0.243	0.118

**Table 2:** Partial Mantel tests of biogeographical distances against climatic and geographical distances for Italian earwigs.

Matrix Correlation		Biogeographical Distances					
Matrix A × Matrix B	Matrix C (Controlling)	Sørensen (βsor)		Simpson (βsim)		Nestedness (βnest)	
		*r*	*p*	*r*	*p*	*r*	*p*
Climatic distances	Centroids	0.380	0.052	0.249	0.108	0.107	0.261
Centroids	Climatic distances	0.376	0.004	0.299	0.017	−0.002	0.555

**Table 3:** Correlation (Pearson coefficient) between latitude and biogeographical similarities of Italian regions with adjacent areas expressed by Sørensen and Simpson indexes.

Adjacent Areas	Sørensen Index		Sørensen Index	
	*r*	*p*	*r*	*p*
Western Europe	0.102	0.698	−0.108	0.680
Central Europe	0.558	0.020	0.334	0.191
Eastern Europe	0.076	0.773	−0.303	0.581
Northern Africa	−0.476	0.054	−0.252	0.332

## Data Availability

Species distribution data are given in Table S1. Geographical and climatic data are available from Fattorini [25].

## Supplementary Materials

The following supporting information can be downloaded at: https://www.mdpi.com/article/10.3390/insects14030235/s1, Table S1: Species distribution.
